# An observational study on the effect of hypercholesterolemia developed after living donor liver transplantation on cardiac event and graft failure

**DOI:** 10.1038/s41598-020-79673-5

**Published:** 2021-01-13

**Authors:** Jungchan Park, Seung-Hwa Lee, Sangbin Han, Ah Ran Oh, Suk-Koo Lee, Gyu-Seong Choi, Myung Soo Park, Keumhee Carriere, Joonghyun Ahn, Gaab Soo Kim

**Affiliations:** 1 Department of Anesthesiology and Pain Medicine, Samsung Medical Center, Sungkyunkwan University School of Medicine, 81 Irwon-ro, Gangnam-gu, Seoul, 06351 South Korea; 2 Division of Cardiology, Department of Medicine, Heart Vascular Stroke Institute, Samsung Medical Center, Sungkyunkwan University School of Medicine, Seoul, South Korea; 3 Department of Surgery, Samsung Medical Center, Sungkyunkwan University School of Medicine, Seoul, South Korea; 4 Department of Medicine, Dongtan Sacred Heart Hospital, Hallym University School of Medicine, Hwaseong, Republic of Korea; 5 Department of Mathematical and Statistical Sciences, University of Alberta, Edmonton, AB Canada; 6 Statistics and Data Center, Samsung Medical Center, Sungkyunkwan University School of Medicine, Seoul, South Korea

**Keywords:** Diseases, Gastroenterology, Medical research, Risk factors

## Abstract

This study sought to evaluate the association between newly-developed significant hypercholesterolemia within one year following living donor liver transplantation (LDLT) and long term outcomes in light of cardiovascular events and graft failure. From October 2003 to July 2017, 877 LDLT recipients were stratified according to development of significant hypercholesterolemia within one year following LDLT. The primary outcome was occurrence of a major adverse cardiac event (MACE), defined as a composite of cardiac death, myocardial infarction, and coronary revascularization after LDLT. The incidence of graft failure, defined as all-cause death or retransplantation, was also compared. A total of 113 (12.9%) recipients developed significant hypercholesterolemia within one year. The differences in incidences of cardiac related events and graft related events began emerging significantly higher in the hypercholesterolemia group after 24 months and 60 months since the LDLT, respectively. After adjustment using the inverse probability of weighting, the hazard ratio (HR) for MACE was 2.77 (95% confidence interval (CI) 1.16–6.61; *p* = 0.02), while that for graft failure was 3.76 (95% CI 1.97–7.17, *p* < 0.001). A significant hypercholesterolemia after LDLT may be associated with cardiac and graft-related outcome; therefore, a further study and close monitoring of cholesterol level after LDLT is needed.

## Introduction

Metabolic disorders including dyslipidemia have been reported to develop more frequently after liver transplantion^1,2^. They are closely related to the onset of cardiovascular events which constitute one of the leading causes of long-term mortality after liver transplantation^3^. Although the link between cholesterol and cardiovascular events in liver transplant recipients has been demonstrated in previous studies^1,2,4,5^, it has not been well established as in the general population. Moreover, the net effect of hypercholesterolemia including graft-related outcome remains uncertain because the liver plays a critical role in cholesterol metabolism. The clinical impact of serum cholesterol in liver transplant recipients may be more complex than the general population^6^.

In end-stage liver disease, serum cholesterol level inversely correlates with disease severity, and lowered cholesterol level has been associated with mortality in decompensated liver disease^7^. The current guideline also states that only limited data suggest potential benefit of statin use in patients with chronic, stable liver disease considering the risk of hepatotoxicity^8^. So, the management of blood cholesterol in liver transplant recipients remains uncertain. In this study, we aimed to evaluate whether newly-developed significant hypercholesterolemia within one year following living donor liver transplantation (LDLT) impacts the onset of cardiovascular events or graft failure of the recipients. Our findings might be helpful for long-term management of liver transplant recipients.

## Results

**Figure 1:**
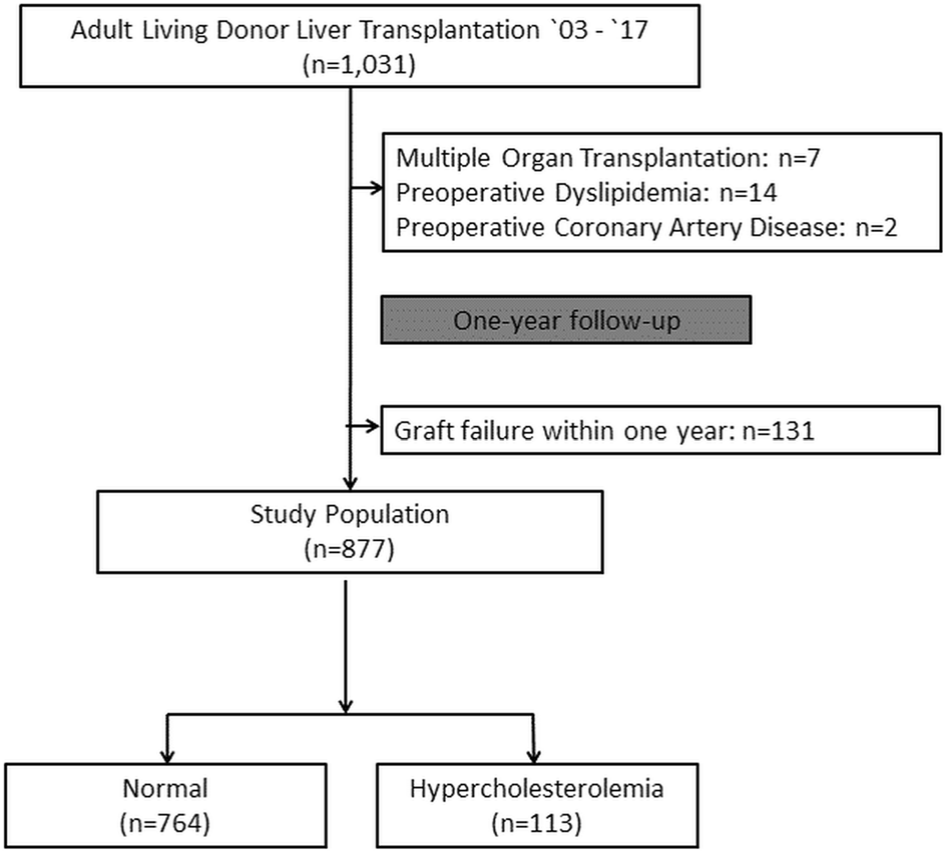
Flowchart of the recipients.

We excluded 7 recipients who underwent multiple organ transplantation, 131 recipients with graft failure within one year after LDLT, 14 recipients with preoperative dyslipidemia or lipid-lowering therapy, and 2 recipients with preoperative coronary artery disease. A total of 877 recipients was left for analysis and divided into two groups: 764 (87.1%) in the normal group and 113 (12.9%) in the hypercholesterolemia group. The flowchart of the recipients is shown in Fig. 1. The hypercholesterolemia group consisted of 98 recipients with serum total cholesterol level greater than 240 mg/dL and 15 recipients with pharmacological treatment for known hypercholesterolemia. The median time interval from LDLT to the first cholesterol measurement was 15 days (interquartile range 13–17 days) in the entire population, and the median period for hypercholesterolemia to be detected was 110 days (interquartile range 61–170 days) in the hypercholesterolemia group. The cholesterol level was increased during the first year after LDLT in the both groups. For the normal group, preoperative cholesterol level was 112.3 (± 45.3) mg/dL and increased to 167.3 (± 41.5) mg/dL postoperatively. Baseline characteristics of the patients are summarized in Table 1. Of note, the use of mechanistic target of rapamycin (mTOR) inhibitors was more frequent in the hypercholesterolemia group (9.7% vs. 17.7%; *p* < 0.02).

**Table 1:** Baseline characteristics.

	Normal (*N* = 764)	Hypercholesterolemia (*N* = 113)	*p* value	SMD	IPW SMD
Preoperative cholesterol level, mg/dL^a^	112.3 (± 45.3)	117.3 (± 45.3)	0.30		
Postoperative cholesterol level, mg/dL^a^	167.3 (± 41.5)	258.3 (± 28.9)			
Male	599 (78.4)	95 (84.1)	0.21	14.6	1.1
Age	52.4 (± 8.3)	52.2 (± 8.7)	0.81	2.4	5.9
Body mass index	24.5 (± 3.4)	24.6 (± 3.4)	0.89	1.4	4.1
Smoking	232 (30.4)	41 (36.3)	0.25	12.6	2.4
Alcohol	264 (34.6)	43 (38.1)	0.53	7.3	3.9
Hepatorenal syndrome	32 (4.2)	7 (6.2)	0.47	9.1	5.8
Encephalopathy	160 (20.9)	23 (20.4)	0.98	1.5	3
SBP	77 (10.1)	13 (11.5)	0.76	4.6	0.1
MELD score	17.5 (± 10.1)	18.0 (± 10.4)	0.61	5	5.7
Total bilirubin	7.80 (± 12.58)	8.48 (± 13.15)	0.59		
INR	1.85 (± 1.19)	1.82 (± 1.02)	0.79		
Creatinine	0.99 (± 0.72)	1.03 (± 0.53)	0.51		
Albumin	3.15 (± 0.63)	3.17 (± 0.65)	0.75	3.2	1.5
**Past medical history**					
Hypertension	82 (10.7)	17 (15.0)	0.23	12.9	< 0.1
Diabetes	133 (17.4)	25 (22.1)	0.28	11.9	0.7
Tuberculosis	34 (4.5)	9 (8.0)	0.17	14.6	2.3
Stroke	5 (0.7)	3 (2.7)	0.12	15.7	0.5
Peripheral artery disease	1 (0.1)	0	> 0.999	5.1	5.1
**Etiology**					
Alcoholic cirrhosis	72 (9.4)	14 (12.4)	0.36	9.5	1.6
Hepatocellular carcinoma	401 (52.5)	60 (52.2)	> 0.999	0.5	0.7
Viral infection	620 (81.2)	87 (77.0)	0.36	10.2	3.6
Acute hepatic failure	61 (8.0)	13 (11.5)	0.28	11.9	1
mTor use	74 (9.7)	20 (17.7)	0.02	23.5	1.7
**Donor factors**					
Age	32.4 (± 11.4)	31.5 (± 11.8)	0.44	7.7	2.3
Macro steatosis, %	6.84 (± 5.87)	6.92 (± 6.19)	0.9	1.3	5.9
GRWR	1.11 (± 0.25)	1.12 (± 0.35)	0.69	3.5	4.6

*SBP* spontaneous bacterial peritonitis, *MELD* model for end-stage liver disease, *INR* international normalized ratio, *GRWR* graft-to-recipient weight ratio.

^a^Pre- and postoperative cholesterol levels were not retained in the statistical adjustment.

**Figure 2:**
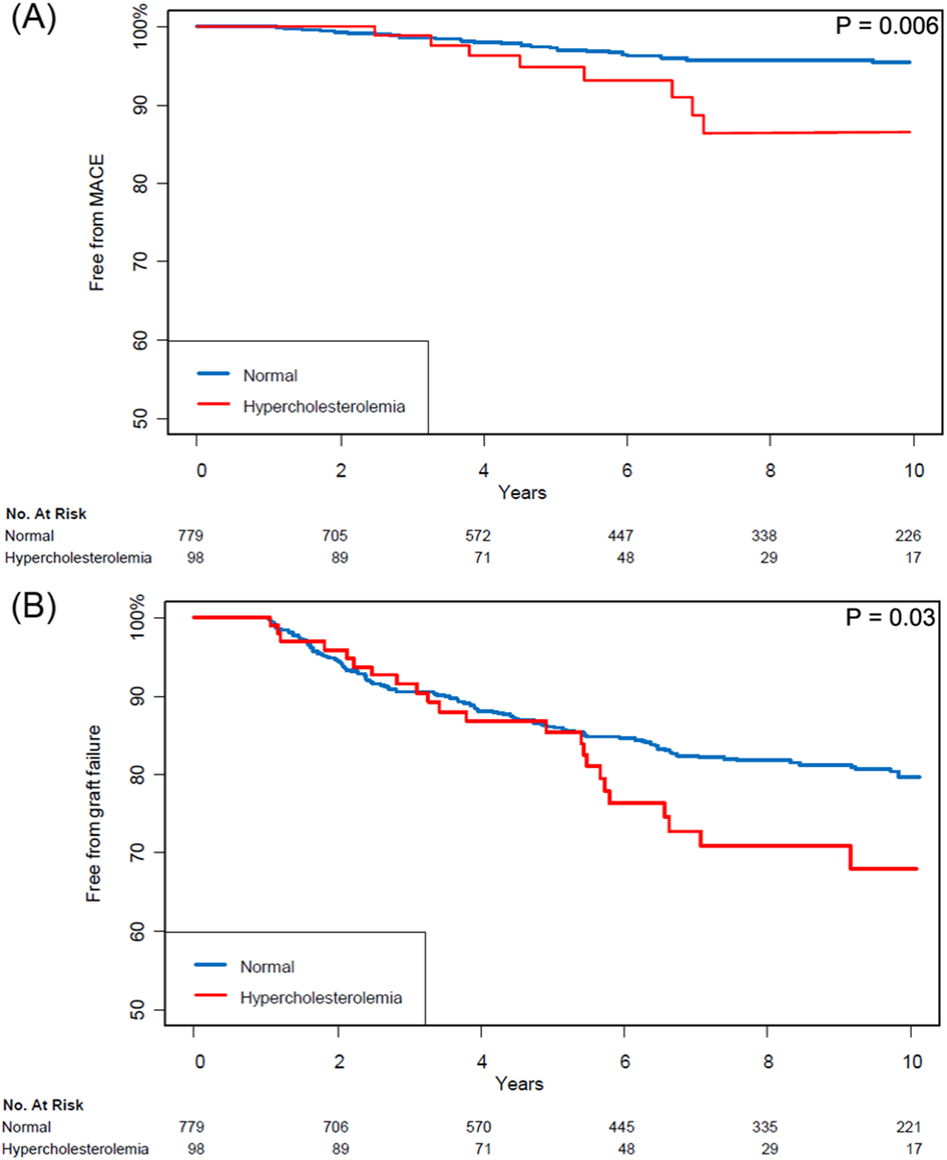
Kaplan–Meier curves of the normal and the hypercholesterolemia groups for (**A**) major adverse cardiac event and (**B**) graft failure.

The median follow-up period was 82 months (interquartile range 46–124 months), and the overall incidences of major adverse cardiac event (MACE) and graft failure were 4.1% and 18.5%, respectively. The incidence of adverse outcomes according to cholesterol in the hypercholesterolemia group level is shown in Supplementary Table S1 and causes of death are summarized in Supplementary Table S2. The Kaplan–Meier curves estimating MACE and graft failure during follow-up period are shown in Fig. 2. Cardiovascular and graft-related outcomes are shown in Tables 2 and 3. To satisfy proportional hazard assumptions for the endpoints, change point analyses were pursued, and the change points were determined to have occurred at 24 months and 60 months after LDLT for cardiac-related and graft-related incidences, respectively. This is consistent with the Kaplan–Meier survival curves in Fig. 2. After adjustment with inverse probability weighting (IPW), the incidences of MACE and graft failure were significantly higher in the hypercholesterolemia group only after the respective change points in time. The cardiac-related incidences were low, occurring later follow-up times. While the hazard before 24 months was not estimable due to few observed events, the hazard after 24 months was higher in the hypercholesterolemia group with hazard ratio (HR) of 2.77 (95% confidence interval (CI) 1.16–6.61; *p* = 0.02) (Table 2). For graft failure, hypercholesterolemia was not significantly associated with the risk for the first 60 months after LDLT (HR 0.94, 95% CI 0.52–1.72; *p* = 0.83), but a significant increased risk emerged after 60 months following LDLT (HR 3.76, 95% CI 1.97–7.17; *p* < 0.001) (Table 3). Postoperative complication of biliary stricture after LDLT and the use of mTOR inhibitor were independently associated with development of significant hypercholesterolemia after LDLT (odds ratio [OR] 2.09, 95% CI 1.39–3.13; *p* < 0.0001 and OR 1.97, 95% CI 1.13–3.41; *p* = 0.02, respectively) (Table 4). In the hypercholesterolemia group, the incidence of adverse outcomes was shown lower in the statin treatment group compared to the non-statin treatment group (Supplementary Table S3).

**Table 2:** Cardiovascular endpoints.

	Normal (*N* = 764)	Hypercholesterolemia (*N* = 113)	Unadjusted HR (95% CI)	*p* value	Adjusted HR (95% CI)	*p* value	IPW adjusted HR (95% CI)	*p* value
**MACE**								
Overall follow-up	26	10	2.91 (1.40–6.05)	0.004	2.96 (1.38–6.32)	0.01	2.78 (1.31–5.92)	0.01
Within 24 months follow-up	6	0						
Beyond 24 months follow-up	20	10	3.87 (1.81–8.29)	< 0.001	4.43 (2.00–9.79)	< 0.001	2.77 (1.16–6.61)	0.02
**Cardiac death**								
Overall follow-up	22	8	2.69 (1.20–6.06)	0.02	2.55 (1.09–5.95)	0.03	2.39 (1.02–5.61)	0.04
Within 24 months follow-up	5	0						
Beyond 24 months follow-up	17	8	3.55 (1.53–8.23)	0.003	3.77 (1.56–9.08)	0.003	1.98 (0.74–5.29)	0.17
**Myocardial infarction**								
Overall follow-up	0	0						
Within 24 months follow-up	0	0						
Beyond 24 months follow-up	0	0						
**Coronary revascularization**								
Overall follow-up	4	2	4.12 (0.75–22.61)	0.1	5.75 (0.96–34.51)	0.06	4.85 (0.92–25.7)	0.06
Within 24 months follow-up	1	0						
Beyond 24 months follow-up	3	2						

Values are n (%), All analyses are others are based on the competing risk analysis.

Covariates include male, age, hypertension, diabetes, tuberculosis, stroke, hepatocellular carcinoma, acute hepatic failure, and mTOR-based immunosuppression.

*MACE* major adverse cardiac event, *HR* hazard ratio, *IPW* inverse probability weighting.

**Table 3:** Graft-related endpoints.

	Normal (*N* = 764)	Hypercholesterolemia (*N* = 113)	Unadjusted HR (95% CI)	*p* value	Adjusted HR (95% CI)	*p* value	IPW adjusted HR (95% CI)	*p* value
**Graft failure**								
Overall follow-up	134	28	1.57 (1.05–2.37)	0.03	1.35 (0.89–2.06)	0.16	1.55 (1.02–2.35)	0.04
Within 60 months follow-up	99	14	0.97 (0.55–1.69)	0.91	0.78 (0.44–1.39)	0.4	0.94 (0.52–1.72)	0.83
Beyond 60 months follow-up	35	14	3.76 (2.01–7.02)	< 0.001	3.82 (2.02–7.22)	< 0.001	3.76 (1.97–7.17)	< 0.001
**All-cause death**								
Overall follow-up	116	25	1.58 (1.03–2.44)	0.04	1.35 (0.86–2.11)	0.19	1.55 (0.99–243)	0.05
Within 60 months follow-up	92	14	1.05 (0.60–1.83)	0.88	0.84 (0.47–1.50)	0.84	1.01 (0.55–1.86)	0.97
Beyond 60 months follow-up	24	11	4.02 (1.96–8.22)	< 0.001	4.16 (1.99–8.68)	< 0.001	4.0 (1.90–8.41)	< 0.001
**Retransplantation**								
Overall follow-up	23	5	1.82 (0.69–4.80)	0.23	1.81 (0.66–4.94)	0.25	1.80 (0.64–5.07)	0.27
Within 60 months follow-up	12	1	0.57 (0.07–4.36)	0.59	0.41 (0.05–3.63)	0.42	0.24 (0.03–1.82)	0.17
Beyond 60 months follow-up	11	4	3.76 (1.18–11.96)	0.02	4.17 (1.29–13.47)	0.02	4.13 (1.26–13.6)	0.02

Values are n (%), All cause death is based on a Cox regression, while the others are based on the competing risk analysis.

Covariates include male, age, hypertension, diabetes, tuberculosis, stroke, hepatocellular carcinoma, acute hepatic failure, and mTOR-based immunosuppression.

Biliary complication was compared only for the overall follow-up considering that proportional hazard assumption was not violated.

*MACE* major adverse cardiac event, *HR* hazard ratio, *IPW* inverse probability weighting.

**Table 4:** Predictors of hypercholesterolemia within 1-year after living donor liver transplantation.

	Unadjusted OR (95% CI)	*p* value	Adjusted OR (95% CI)	*p* value
Male	0.69 (0.40–1.17)	0.17	1.43 (0.83–2.45)	0.20
Age	1.00 (0.97–1.02)	0.8	0.99 (0.96–1.01)	0.36
Smoking	0.77 (0.51–1.16)	0.21		
Alcohol	0.86 (0.57–1.29)	0.47		
Hepatorenal syndrome	1.51 (0.65–3.51)	0.34		
Encephalopathy	0.97 (0.59–1.58)	0.89		
SBP	1.16 (0.62–2.17)	0.64		
MELD score	1.00 (0.99–1.02)	0.67		
Total bilirubin	1.00 (0.9–1.02)	0.57		
INR	0.98 (0.82–1.17)	0.83		
Creatinine	1.09 (0.85–1.41)	0.49		
Alb	1.05 (0.77–1.44)	0.75		
**Past medical history**				
Hypertension	1.47 (0.84–2.59)	0.18	1.56 (0.87–2.80)	0.14
Diabetes	1.35 (0.83–2.18)	0.23		
Tuberculosis	1.86 (0.87–3.99)	0.11	1.94 (0.88–4.26)	0.10
Stroke	4.14 (0.98–17.57)	0.054	4.17 (0.94–18.53)	0.06
**Etiology**				
Alcoholic cirrhosis	1.36 (0.74–2.50)	0.32		
Hepatocellular carcinoma	0.99 (0.67–1.47)	0.96		
Viral infection	0.78 (0.48–1.25)	0.3		
Acute hepatic failure	1.50 (0.79–2.83)	0.21		
mTor-based immunosuppression	2.00 (1.17–3.44)	0.01	1.97 (1.13–3.41)	0.02
Biliary leakage	1.00 (0.55–1.81)	0.99		
Biliary stricture	2.07 (1.39–3.09)	< 0.001	2.09 (1.39–3.13)	< 0.001
**Donor**				
Age	0.99 (0.98–1.01)	0.44		
Macro steatosis, %	1.00 (0.97–1.03)	0.99		
GRWR	1.16 (0.56–2.40)	0.69		

*SBP* spontaneous bacterial peritonitis, *MELD* model for end-stage liver disease, *INR* international normalized ratio, *GRWR* graft-to-recipient weight ratio, *OR* odds ratio.

**Figure 3:**
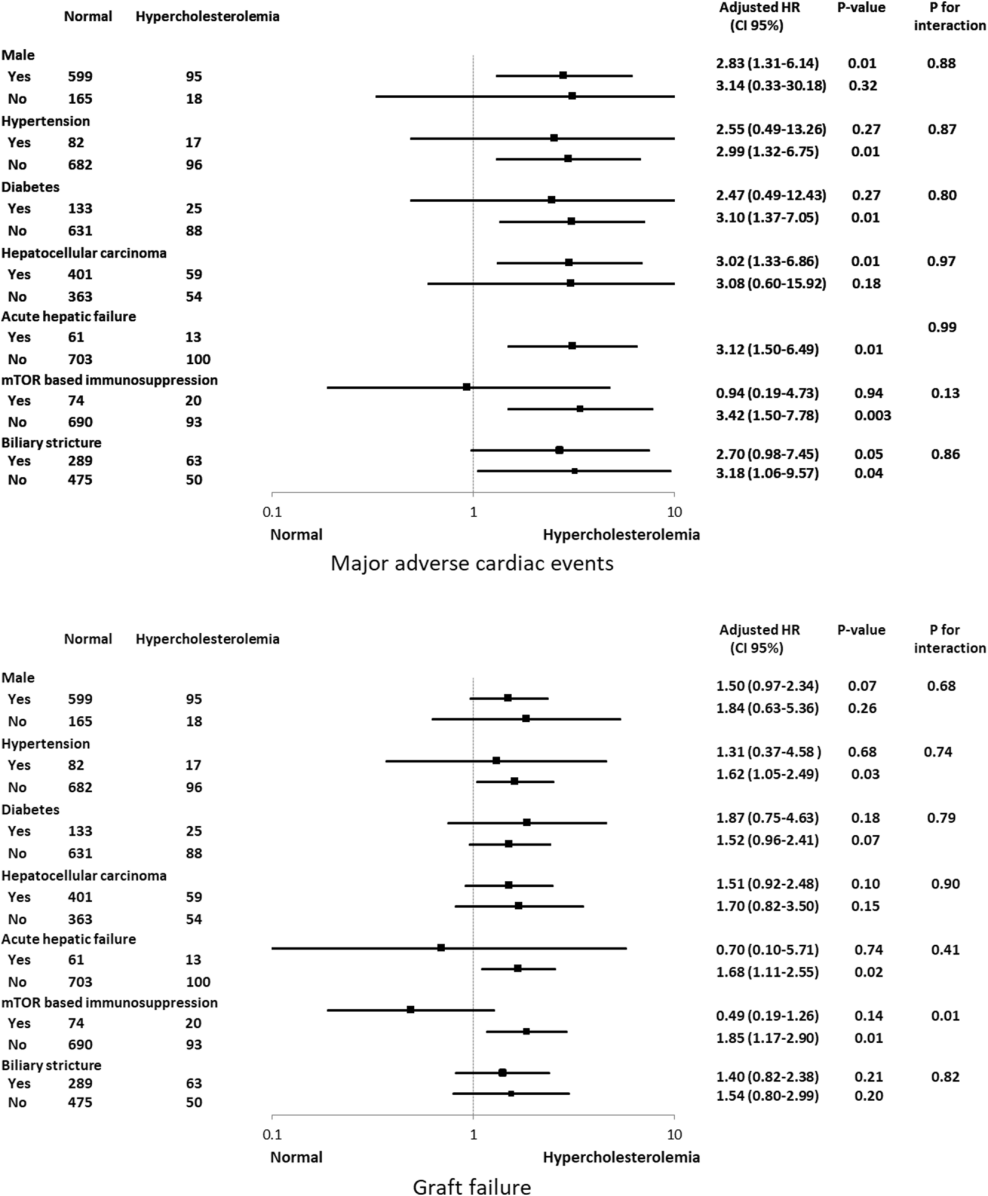
Subgroup analyses.

**Figure 4:**
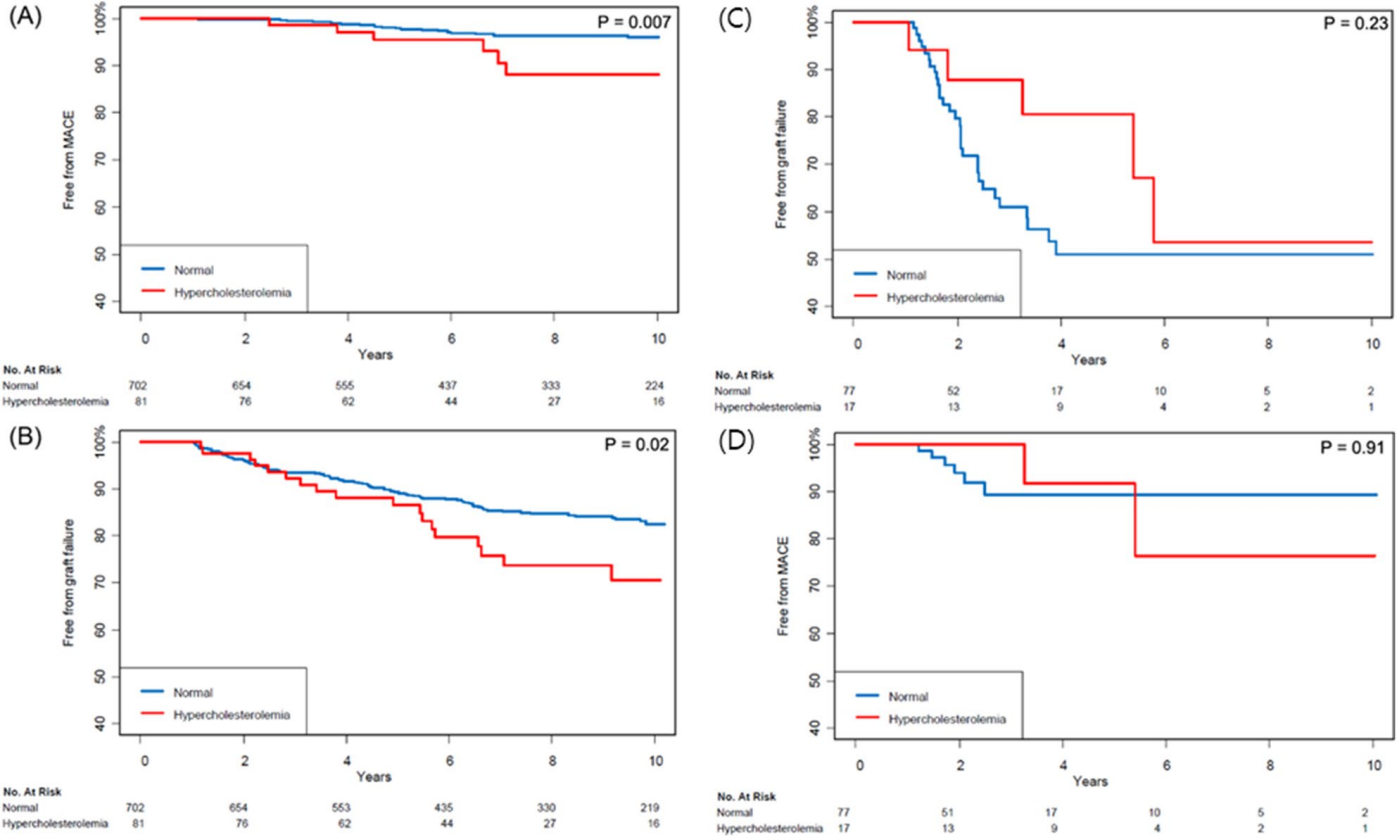
Kaplan–Meier curves for (**A**) major adverse cardiac event and (**B**) graft failure in recipients without the use of mechanistic target of rapamycin and (**C**) major adverse cardiac event and (**D**) graft failure in recipients with the use of mechanistic target of rapamycin.

In the subgroup analysis, we determined whether the incidence of MACE or graft failure was affected by other covariates by calculating HR in various complex subgroups (Fig. 3). The incidence of MACE did not show a significant interaction with any variables. For graft failure, hypercholesterolemia was significantly associated with increased risk in recipients without mTOR therapy (HR 1.85, 95% CI 1.17–2.90, *p* = 0.01), while it showed a marginally insignificant association with decreased risk of graft failure in recipients with mTOR therapy (HR 0.49, 95% CI 0.19–1.26, *p* = 0.14), and the interaction had a *p*-value = 0.01. The Kaplan–Meier curves estimating MACE and graft failure in the subgroups according to the use of mTOR therapy are shown in Fig. 4.

## Discussion

In this study, development of significant hypercholesterolemia within one year after LDLT was significantly associated with cardiovascular events, and it was also associated with graft failure after long-term follow-up. The use of mTOR inhibitors, which were selectively used in recipients with advanced stages of hepatocellular carcinoma, independently increased the development of significant hypercholesterolemia. Recently, there has been a growing interest in long-term management of liver transplant recipients, and these findings suggest that significant hypercholesterolemia after LDLT may need to be further evaluated and properly treated.

The incidence of hypercholesterolemia in liver transplant recipients has consistently been reported to be higher compared to the general population^1,2,4^. In liver transplantation, gene transmission by the graft liver could result in a gene mutation causing hypercholesterolemia^9^, and immunosuppressive treatments or cholestasis afterward could also induce hypercholesterolemia^4,10^. In this study, 12.9% (113/877) of recipients developed a new significant hypercholesterolemia within one year after LDLT. The incidences of hypercholesterolemia and cardiovascular events are similar to those reported from LDLT in Asian countries^2^, but seem relatively lower compared to Western countries^1,2,11,12^. This may be related to the fact that disease etiology as well as indications for or graft types of liver transplantation between Eastern and Western countries are discrete^13^. The low incidence of cardiovascular events may be due to the small portion of high-risk underlying etiologies such as nonalcoholic fatty liver disease^1^, but whether the differences in donor type or ethnicity affect the development of hypercholesterolemia remains uncertain.

The link between hypercholesterolemia and cardiovascular events has been demonstrated in previous studies, mostly from deceased donor-type liver transplant^14^. In this study, we only recruited recipients who underwent right-lobe LDLT, because the difference in type or size of the graft liver is related to post-transplant cholesterol metabolism which may affect long-term outcomes. During the long-term follow-up, we compared both graft-related and cardiovascular outcomes, because clinical situations for graft-related outcomes may be more complex than cardiovascular outcomes, considering that the liver is the primary site of cholesterol biosynthesis and storage. Under a normal physiologic state, it excretes cholesterol in the form of bile acid via the biliary tract, and so hepatic dysfunction could result in an impaired cholesterol metabolism pathway depending on the severity of the disease^15,16^. Conversely speaking, liver transplantation could reverse this impairment by improving hepatic dysfunction^17^, suggesting that changes in cholesterol metabolism after liver transplantation could be indicators of graft function^6^. Furthermore, perioperative cholesterol level could also affect graft-related outcomes of liver transplant recipients, because a sufficient cholesterol supply is important in liver regeneration^18^. In this study, hypercholesterolemia was associated with graft failure only after long-term follow-up, but the exact pathway remains unknown.

Another interesting finding was that the incidence of biliary stricture as a complication after LDLT was higher in the hypercholesterolemia group. Biliary stricture after liver transplantation may lead to cholestasis which impairs the intestinal absorption of all types of sterols. So, this could be related to the mechanism of hypercholesterolemia^6^. In addition, biliary stricture was shown to be significantly associated with the development of hypercholesterolemia in the multivariable analysis. However, further studies are needed because the changes in cholesterol metabolism are not correctly mirrored by any serum measurement^19^.

The use of mTOR inhibitors was also found to be associated with the development of significant hypercholesterolemia in agreement with previous studies^10,20^. Despite pronounced side effect of dyslipidemia, there is a growing body of evidence that the net effect of mTOR inhibitors may be cardioprotective^20^. In animal studies, mTOR inhibition was demonstrated to improve endothelial function, inhibit smooth muscle cell proliferation, and decrease macrophage content in the plaque^21^. Cholesterol efflux induced by mTOR inhibition also decreases lipid accumulation in the plaque^20,22^. In humans, an anti-atherogenic effect was presented in heart transplant recipients, and the drug was shown to be cardioprotective^23^. However, our subgroup analysis showed that hypercholesterolemia was significantly associated with graft failure only in recipients without mTOR inhibition, but no significant interaction was observed for the use of mTOR with the association between hypercholesterolemia and MACE. This result may be related to the fact that the recipients on mTOR inhibition were those with an advanced hepatoceulluar carcinoma, suggesting that hypercholesterolemia may be associated with graft failure only in an advanced hepatoceulluar carcinoma, but it may not affect the association between hypercholesterolemia and MACE. In addition, this interaction with mTOR inhibition may also suggest that, with the cardioprotective effects of mTOR inhibitors, sufficient cholesterol biosynthesis after transplantation may indicate or contribute to improved graft function.

There is no definite recommendation or guidelines that are currently available pertaining to blood cholesterol management in liver transplant recipients, and the current guideline states that, although statin is not contraindicated in stable liver disease, supporting evidence for its potential benefit is limited^8^. In addition to lipid-lowering effect, the use of statin has shown to inhibit hepatocellular carcinoma recurrence^24^ and reduce mortality of chronic liver disease by preventing hepatic decompensation and the progression of hepatic fibrosis^25^ In the present study, the incidence of adverse events was numerically lower in the recipients on statin therapy, but the number of recipients on statin therapy was too low to be properly analyzed. Therefore, larger registries or randomized trials are needed to accurately evaluate the benefit of statin therapy in liver transplant recipients. Meanwhile, cardiovascular risk assessment in liver transplant recipients should be detailed and individualized, because increased cholesterol after liver transplantation is not necessarily all atherogenic^6^. For instance, cholestasis leads to formation of lipoprotein X which is nonatherogenic, but it is frequently mistaken as atherogenic lipid on routine tests and leads to unnecessary prescription of statin resulting in an accumulation to a toxic level^26^.

This study has limitations. They include the nature of a nonrandomized and observational study, in which the results might have been affected by confounding factors. Although an IPW analysis was performed to adjust for these potential confounding factors, unmeasured variables were not able to be corrected. Absence of a detailed lipid profile including low-density lipid, high-density lipid, and lipoprotein X is another limitation. A separate analysis on atherogenic or nonatherogenic lipids might show different results. Lastly, despite the use of a standard institutional protocol, the time intervals between follow-up examinations may differ among the recipients, and the incidence of graft dysfunction could not be compared. In additions, details of the institutional protocol for patient managements have changed during the long study period. Despite these limitations, our study demonstrated a link between significant hypercholesterolemia and cardiovascular outcome in LDLT recipients.

## Methods

### Study population and data collection

The study protocol was approved by the Institutional Review Board at Samsung Medical Center (No. 2018-12-095-002) and was conducted in accordance with the principles of the Declaration of Helsinki. We used liver transplantation database of Samsung Medical Center which is not an open access. From October 2003 to July 2017, a consecutive 1031 adult recipients of right-lobe LDLT were initially enrolled in our registry. The exclusion criteria were: (1) recipients with multiple organ transplantation; (2) recipients with follow-up loss or graft failure within 1 year after LDLT; (3) recipients who preoperatively had dyslipidemia or were on lipid-lowering therapy, and (4) recipients who preoperatively had coronary artery disease. Clinical, laboratory, and outcomes data were independently collected by a trained study coordinator using a standardized case report form and protocol. All recipients were included anonymously after deidentification. The need for individual consent was waived by the Institutional Review Board at Samsung Medical Center.

### Definition and outcomes

Significant hypercholesterolemia was defined as serum total cholesterol level greater than 240 mg/dL or pharmacological treatment for known hypercholesterolemia^8^. Hypertension was defined as either self-reported or systolic blood pressure > 140 mmHg. Resting blood pressure was measured when patients were admitted. Diabetes mellitus was defined as history of type 1 or type 2 diabetes mellitus, treated either pharmacologically or through dietary changes.

The primary endpoint was occurrence of a MACE, defined as the composite of cardiac death, myocardial infarction, and coronary revascularization either by intervention or operation during follow-up period. Any death was considered to be of cardiac origin unless a definite non-cardiac cause could be established^27^. Myocardial infarction was defined as recurrent symptoms with new electrocardiographic changes compatible with myocardial infarction or cardiac marker elevation according to the Fourth Universal Definition^28^. The secondary endpoint was graft failure, defined as all-cause death or retransplantation, and biliary complications consisting of biliary leakage and stricture were also compared. Clinical outcomes during the overall follow-up period were compared.

### Anesthetic and surgical management

The standardized anesthetic and surgical management protocols of our institution have been described elsewhere^29^. After applying standard monitoring devices (i.e., peripheral capillary oxygen saturation, five-lead electrocardiogram, and noninvasive arterial blood pressure), general anesthesia was induced with thiopental sodium (5 mg/kg) and maintained with isoflurane. Remifentanil was infused up to 0.20 μg/kg/min in response to hemodynamic changes. Intravenous fluids and pressor drugs such as norepinephrine, vasopressin, and dopamine were infused to maintain mean arterial pressure of 70 mmHg or more.

All grafts consisted of segments 5 through 8 of Couinaud’s classification. Parenchyma transection was performed using an ultrasonic dissector and a bipolar coagulator. Intermittent hepatic inflow occlusion was used to minimize blood loss during parenchymal resection. Five minutes after intravenous heparin (5000 U) injection, the graft liver was removed and flushed with histidine-tryptophan-ketoglutarate solution. The graft was then implanted using the piggyback technique. After portal vein anastomosis, the hepatic vein and portal vein were unclamped for reperfusion. Following reperfusion, segments 5 and 8 veins were anastomosed to the inferior vena cava and hepatic artery, respectively, and biliary anastomosis were then performed.

### Postoperative and immunosuppressive management

Recipients were closely monitored in the intensive care unit for at least the first 48 h after LDLT. Routine blood tests were done daily during the hospital stay. Attempts for early detection of postoperative complications such as bleeding, thrombosis, biliary stenosis, or biliary leakage were also made at the intensive care unit. When abdominal drainage revealed biliary leakage, or biliary stricture was suspected with elevated bilirubin after postoperative day 4, ultrasonography was initially performed and then confirmed by retrograde cholangiography. Nonsurgical intervention such as drainage was primary choice for biliary complications.

Follow-up blood tests after discharge were performed during visits to the outpatient department. A first visit to the outpatient department was recommended at two weeks after discharge, and monthly visits were recommended for the first year after LDLT. After one year of follow-up, routine visits in every two months to the outpatient department were encouraged. Cardiac evaluation and lipid managements of recipients followed the current guidelines^30,31^. Recipients with cardiac symptoms were referred to cardiologists for proper evaluation, and for those with hypercholesterolemia, statin was prescribed according to the guidelines^30,31^.

Immunosuppression was based on a quadruple regimen: induction with methylprednisolone plus basiliximab and maintenance with tacrolimus plus mycophenolate mofetil starting on the third postoperative day. The plasma concentration of tacrolimus was titered at 10–15 ng/mL. The use of mTOR inhibitor was adopted in 2013, and it was used for recipients with hepatocellular carcinoma beyond Milan criteria or those with alpha fetoprotein over 200 ng/mL. In recipients with the use of mTOR inhibitors, mycophenolate mofetil was tapered, and tacrolimus level was decreased.

### Statistical analysis

Continuous variables of each group were compared using the t-test or the Wilcoxon rank-sum test where applicable and presented as mean ± standard deviation. Categorical variables were evaluated using Chi-square or Fisher’s exact test. Kaplan–Meier estimates were used to construct survival curves and compared using the log-rank test. Covariates with a univariate effect with a *p* value < 0.2 or that were clinically relevant were initially considered in the multivariable logistic regression model before being reduced to identify significant factors. Adjustments were made with the following baseline variables: male, age, hypertension, diabetes mellitus, tuberculosis, stroke, hepatocellular carcinoma, acute hepatic failure, and mTOR inhibitor usage. To study the influence of hypercholesterolemia on the long term clinical outcomes following LDLT, we first compared their baseline characteristics. Although there did not appear to be any imbalance between the two groups, we advocated the propensity score method and conducted a rigorous adjustment for differences in baseline characteristics of patients using weighted Cox proportional-hazards regression models with the stabilized IPW method using the propensity scores. The propensity scores were estimated using multiple logistic regression model to predict hypercholesterolemia on all baseline characteristics listed in Table 1. Balance was deemed to be achieved when the standard mean difference between the two groups is within 20% and the ratio of variance is near 1.0 for each covariate. The reduction in risk of outcomes was compared using the Cox regression model or competing risk model, verifying proportional hazard assumptions. When the assumption was violated for each endpoint, we checked the feasibility for time-dependent coefficients of the group over time. The change point was estimated as the time that maximizes the log likelihood function, and then deliberated its clinical relevance before adopting it. We also performed a subgroup analysis to reveal hidden interactions with male, hypertension, diabetes mellitus, hepatocellular carcinoma, acute hepatic failure, biliary stricture, and mTOR inhibitor usage. Statistical analyses were performed with R 3.5.0 (R core team, 2018) and SAS 9.4 (SAS Institute Inc., Cary, NC, USA). All tests were two-tailed, and a *p* value < 0.05 was considered statistically significant.

### Ethical approval

Institutional Review Board (No. 2018-12-095-002).

## Conclusion

Development of significant hypercholesterolemia within 1 year after LDLT appears to be associated with cardiovascular and graft-related outcome, and therefore should be cautiously monitored and managed. The beneficial effect of statin in those recipients needs further evaluation.

## Supplementary Information

Supplementary Information.

## Data Availability

The data underlying this study contain sensitive information and cannot be made publicly available. Interested researchers can submit data access requests.

## Abbreviations

LDLT: Living donor liver transplantation
MACE: Major adverse cardiac event
HR: Hazard ratio
OR: Odds ratio
IPW: Inverse probability weighting
