# Supplementary material for: An observational study on the effect of hypercholesterolemia developed after living donor liver transplantation on cardiac event and graft failure
Source: Sci Rep. 2021 Jan 13;11:959. doi: 10.1038/s41598-020-79673-5 (PMC7806822; doi:10.1038/s41598-020-79673-5)
Supplement: Supplementary file 1 — Supplementary Information. [file 41598_2020_79673_MOESM1_ESM.docx]

**An observational study on the effect of hypercholesterolemia developed after living donor liver transplantation on cardiac event and graft failure**

Jungchan Park, MD^1^, Seung-Hwa Lee, MD^2^, Sangbin Han, MD^1^, Ah Ran Oh, MD^1^, Suk-Koo Lee, MD, PhD^3^, Gyu-Seong Choi, MD^3^, Myung Soo Park, MD^4^, Keumhee Carriere, PhD^5,6^, Joonghyun Ahn, PhD^6^ & Gaab Soo Kim, MD, PhD^1^

^1^Department of Anesthesiology and Pain Medicine, Samsung Medical Center, Sungkyunkwan University School of Medicine, Seoul, Korea

^2^Division of Cardiology, Department of Medicine, Heart Vascular Stroke Institute, Samsung Medical Center, Sungkyunkwan University School of Medicine, Seoul, Korea

^3^Department of Surgery, Samsung Medical Center, Sungkyunkwan University School of Medicine, Seoul, Korea

^4^Department of Medicine, Dongtan Sacred Heart Hospital, Hallym University School of Medicine, Republic of Korea

^5^Department of Mathematical and Statistical Sciences, University of Alberta, Edmonton, AB, Canada

^6^Statistics and Data Center, Samsung Medical Center, Sungkyunkwan University School of Medicine, Seoul, Korea

J.C.P and S.H.L contributed equally to this work.

**Keywords:** living donor liver transplantation, hypercholesterolemia, clinical outcome

**Contact Information**

Gaab Soo Kim, MD, Department of Anesthesiology and Pain Medicine, Samsung Medical Center, Sungkyunkwan University School of Medicine, 81 Irwon-ro, Gangnam-gu, Seoul, Korea, 06351

Tel: +82-2-3410-0360; Fax: +82-2-3410-0361; E-mail address: gskim@skku.edu

**Supplemental table 1.** Clinical outcomes of the hypercholesterolemia group according to peak cholesterol level

|  | **MACE** | **Graft failure** |
| --- | --- | --- |
| Peak cholesterol level, mg/dL |  |  |
| 240-300 (n=107) | 10 (9.3) | 26 (24.3) |
| 300-400 (n=5) | 0 | 2 (40.0) |
| >400 (n=1) | 0 | 0 |

MACE, major adverse cardiac event

**Supplemental table 2.** Causes of death.

|  | **Normal (*N* = 764)** | **Hypercholesterolemia (*N* = 113)** |
| --- | --- | --- |
| Cardiovascular | 1 (0.1) | 0 |
| Hepatic failure | 17 (2.2) | 5 (4.4) |
| Recurrence of hepatocellular carcinoma | 55 (7.2) | 7 (6.2) |
| Other cancer | 10 (1.3) | 0 |
| Pulmonary | 8 (1.0) | 6 (5.3) |
| Renal | 1 (0.1) | 0 |
| Other undefined | 23 (3.0) | 7 (6.2) |

**Supplementary Table 3.** Clinical outcomes of the hypercholesterolemia group according to statin use

|  | **No statin (N = 98)** | **Statin (N = 15)** | ***p* value** |
| --- | --- | --- | --- |
| MACE | 9 (9.2) | 1 (6.7) | >0.99 |
| Cardiac death | 7 (7.1) | 1 (6.7) | >0.99 |
| Myocardial infarction | 0 | 0 |  |
| Coronary revascularization | 2 (2.0) | 0 |  |
| Stroke | 1 (1.0) | 0 |  |
| Graft failure | 25 (25.5) | 3 (20) | 0.76 |
| Death | 23 (23.5) | 2 (13.3) | 0.52 |
| Retransplantation | 3 (3.1) | 2 (13.3) | 0.13 |

MACE, major adverse cardiac event
